# Effects of Different Temperatures on the Chemical Structure and Antitumor Activities of Polysaccharides from *Cordyceps militaris*

**DOI:** 10.3390/polym10040430

**Published:** 2018-04-12

**Authors:** Eliyas Nurmamat, Hongxing Xiao, Yan Zhang, Ziwei Jiao

**Affiliations:** 1 College of Life Sciences, Northeast Normal University, Changchun 130024, China; nuermaimaitiyili@126.com; 2 College of Biological and Geographic Sciences, Yili Normal University, Yining 835000, China; jihaiyu1247@163.com; 3 Key Laboratory of Food Nutrition and Safety, Ministry of Education, School of Food Engineering and Biotechnology, Tianjin University of Science and Technology, Tianjin 300457, China

**Keywords:** *C. militaris*, extraction temperature, polysaccharides, physicochemical properties, antitumor activity

## Abstract

The effects of different extraction temperatures (4 and 80 °C) on the physicochemical properties and antitumor activity of water soluble polysaccharides (CMPs-4 and CMPs-80) from *Cordyceps militaris (C. militaris)* were evaluated in this study. The results of gas chromatography (GC) and high-performance gel permeation chromatography (HPGPC) showed that a higher extraction temperature could degrade the polysaccharides with 188 kDa, mainly composed of glucose, and increase the dissolution rate of polysaccharides about 308 kDa, mainly consisting of rhamnose and galactose. In addition, the CMPs displayed the same sugar ring and category of glycosidic linkage based on Fourier-transform infrared spectroscopy (FTIR) analysis, however, their invisible structural difference occurred in the specific rotation and conformational characteristics according to the results of specific optical rotation measurement and Congo red test. In vitro antitumor experiments indicated that CMPs-4 possessed stronger inhibitory effects on human esophagus cancer Eca-109 cells by inducing cell apoptosis more than CMPs-80 did. These findings demonstrated that the polysaccharides extracted with cold water (4 °C) could be applied as a novel alternative chemotherapeutic agent or dietary supplement with its underlying antitumor property.

## 1. Introduction

*C. militaris*, an entomogenous fungus belonging to the class *Ascomycetes*, has been widely used as a folk tonic food and therapeutic drug for various diseases in East Asia [1]. *C. militaris*, known as the Chinese rare caterpillar fungus, has a similar pharmacological effect to the well-known Chinese traditional fungus *Cordyceps sinensis* [2]. Although a variety of bioactive components, such as cordycepic acid, cordycepin, adenine, adenosine, etc., have been reported, the polysaccharides are usually regarded as one of the most abundant bioactive substances, with the highest content in *C. militaris* [3,4]. Modern research has shown that polysaccharides from *C. militaris* possess multiple biological properties, such as antioxidant [5,6,7], antitumor [8,9], immunoregulatory [10,11,12], anti-hyperlipidemic [13], anti-inflammatory activities [14], etc.

In general, the physicochemical properties of polysaccharides using conventional water extraction were strongly affected by multiple parameters, such as the source of raw materials, water temperature, extraction time, extraction solvent, ratio of liquid to solid, and so forth [15,16]. In addition, previous research has shown that different extraction methods influence the structures and pharmacological activities of polysaccharides [17]. Zhu et al. compared the influence of various extraction methods on the chemical structure and antitumor activity of *Cordyceps gunnii mycelia* polysaccharides, and their remarkable differences were observed in their antitumor effects and physicochemical properties, such as scanning electron microscopy, intrinsic viscosity, and specific rotation [18]. Zhang et al. revealed that ultrasonic treatments may induce the degradation of polysaccharides and further changes in their chemical structure, thus resulting in poor antioxidant capability [19]. Zhao et al. reported that a higher extraction temperature and longer extraction time could cause the hydrolysis of polysaccharides, and further lead to the reduction of polysaccharide yield and the variation of microstructure [20]. Therefore, tremendous efforts should be made to explore extraction technologies for the strongest biological activities of polysaccharides.

Recently, researches concerning the *C. militaris* polysaccharide have mainly focused on the optimization of extraction conditions for maximum yield and good bioactivity, however, the influence of extraction temperature on the physicochemical properties and antitumor activities of polysaccharides remains limited. In this study, the main objective was to investigate the effects of cold- and hot-water extraction from *C. militaris* on the relationship between the structural characterization and antitumor activities of polysaccharides, and to further evaluate their antitumor capacities by comparing the in vitro inhibition effects on human esophagus cancer Eca-109 cells. The obtained CMPs were named CMPs-4 and CMPs-80, respectively, according to their extraction temperature (4 and 80 °C). 

## 2. Results

### 2.1. Chemical Properties and Monosaccharide Composition

**Figure 1:**
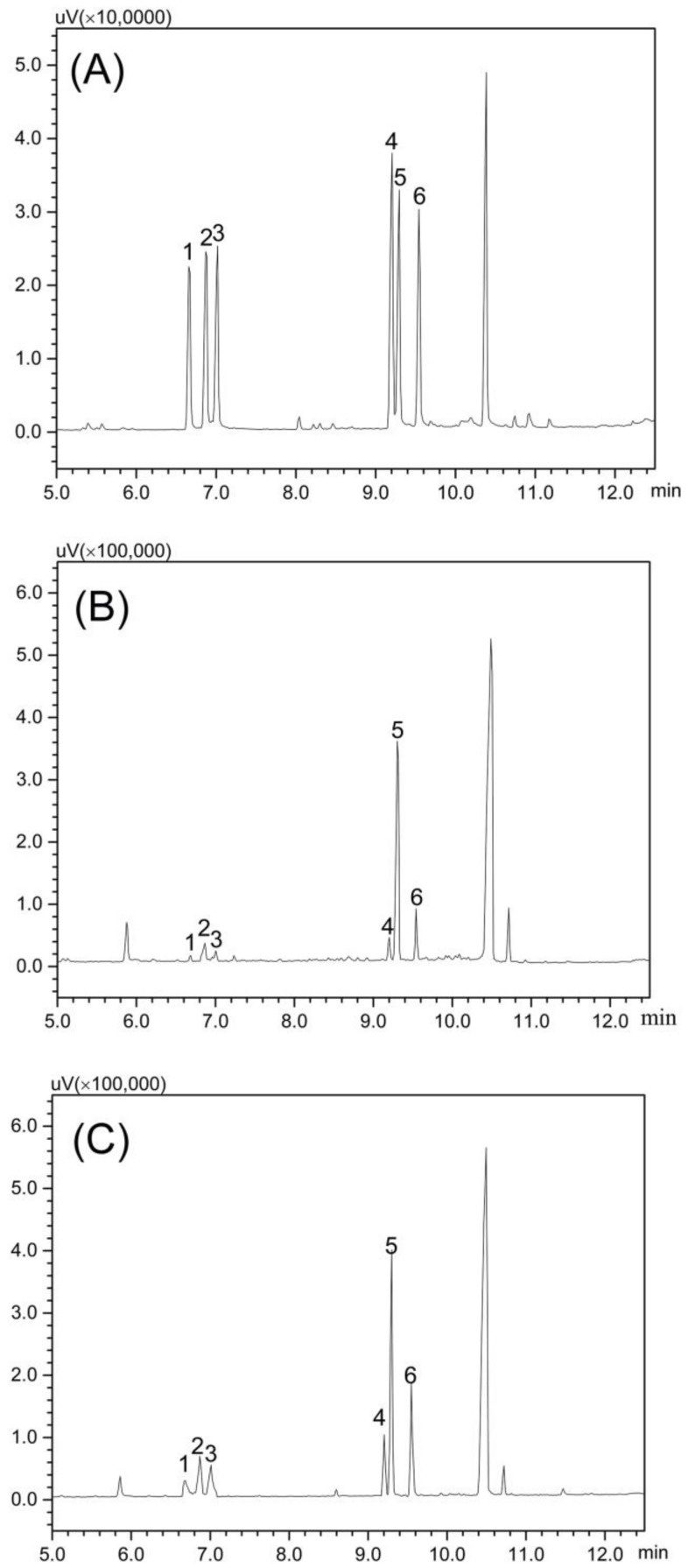
gas chromatography (GC) spectra of standard monosaccharides (**A**), CMPs-4 (**B**), and CMPs-80 (**C**). Peak identity: Rhamnose (1), Arabinose (2), Xylose (3), Mannose (4), Glucose (5), and Galactose (6).

The sugar and protein contents, and their molar ratio of polysaccharide samples, are shown in Table 1. The proportions of total sugar in CMPs-4 and CMPs-80 could reach 87.63% and 85.42%, respectively, and little protein was detected. The monosaccharide composition of CMPs was measured and identified by comparing its retention time with that of standard monosaccharides using GC analysis (Figure 1). Both of them were mainly composed of rhamnose, arabinose, xylose, mannose, glucose, and galactose, in molar ratios of 0.24:0.57:0.48:1.00:12.41:1.63 and 3.98:0.62:0.42:1.00:6.70:3.18, respectively. The results showed that CMPs-4 and CMPs-80 had similar monosaccharide constituents and a higher proportion of glucose in CMPs-4, while the opposite trend was found in the proportions of rhamnose and galactose, indicating that a higher extraction temperature could degrade the polysaccharide mainly composed of glucose and increase the dissolution rate of polysaccharides consisting of rhamnose and galactose.

### 2.2. Molecular Weight Distribution

The regression equation of the standard dextran curve was established as follows: *y* = −0.3866*x* + 8.8472, *R*^2^ = 0.9904 (*y* = lg*M*_w_, *x* = *R*_t_). The retention time, molecular weight population, and relative contents of CMPs-4 and CMPs-80 are displayed in Table 2. The HPGPC profiles of CMPs-4 and CMPs-80 presented two peaks, indicating that the polysaccharide fractions of the two samples contained two major molecular weight distributions. A low molecular weight (approximately 2.5 kDa) was observed with different relative contents in both CMPs. However, a higher molecular weight (about 308 kDa) with a higher percentage area (62.46%) was detected in CMPs-80, compared to the lower molecular weight (about 188 kDa) with a lower percentage area (53.23%) detected in CMPs-4. This suggested that a higher extraction temperature might result in material cell disruption and further speed up the diffusion of macromolecular substances into water. Therefore, a different temperature treatment during the extraction process could influence the conformation of polysaccharides and change the distribution of polysaccharide compositions.

### 2.3. IR Spectral Characteristics

**Figure 2:**
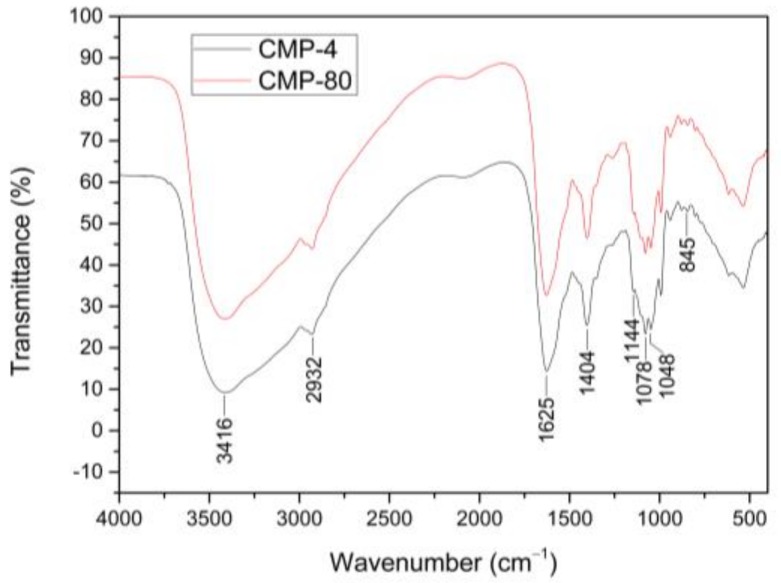
The IR spectras of CMPs-4 and CMPs-80.

IR spectra of CMPs extracted with different temperatures showed very similar absorption bands (Figure 2). Three characteristic absorption bands of the polysaccharides were displayed in the IR spectrums: a broad band at around 3416 cm^−1^ attributed to O–H stretching vibrations, a weak band at around 2932 cm^−1^ due to C–H stretching vibrations, and a band at around 1404 cm^−1^ attributed to the C–H bending vibrations. In addition, the strong absorption band at around 1625 cm^−1^ was derived from C=O stretching vibrations. The absorption bands in the range of 1144–1048 cm^−1^ could be assigned to the stretching and bending vibrations of C–O, C–C and C–O–C, and C–O–H, indicating the presence of pyranose rings, and the band at around 845 cm^−1^ suggested the presence of α-type glycosidic linkages in CMPs. This observation was consistent with the results of Zhu et al. [21], who indicated that the polysaccharides (CMPS-II and CBPS-II), isolated from the cultivated fruiting body and mycelium of *C. militaris*, mainly contained mannose, glucose, and galactose with α-type glycosidic linkages. The IR spectrums of CMPs-4 and CMPs-80 were almost identical only with a slight difference in the intensity of bands, indicating that different extraction temperatures had no distinct effect on sugar rings or the category of glycosidic linkages of polysaccharides, which coincided with the analysis of monosaccharide composition.

### 2.4. Specific Optical Rotation Analysis of Polysaccharides

The specific optical rotations of CMPs-4 and CMPs-80 were +49° and +52°, respectively, which showed the presence of α-glycosidic bonds in these two polysaccharides. The difference in specific optical rotations for both CMPs-4 and CMPs-80 also indicated that different extraction temperatures could cause changes in the stereochemical structure of polysaccharides, which might influence the strength of bioactivities. 

### 2.5. Conformational Characteristics of Polysaccharides

**Figure 3:**
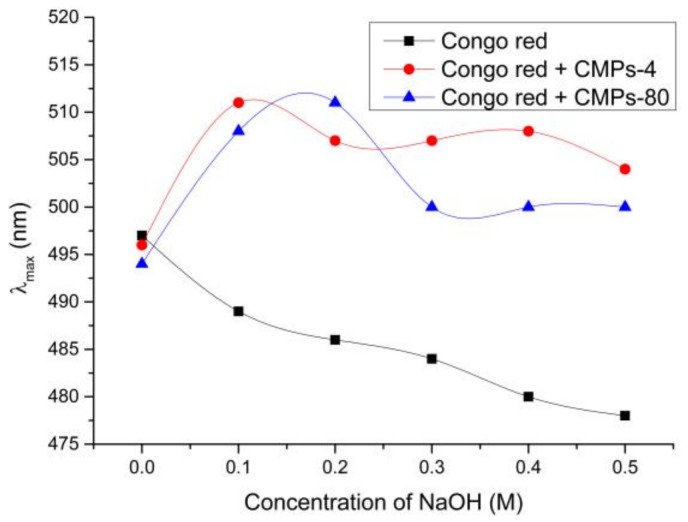
Conformational characteristics of CMPs-4 and CMPs-80.

Congo red, an acid dye, can interact with polysaccharides with triple-helical conformations to form complexes, leading to a bathochromic shift of the absorption maximum [22]. Figure 3 displays the maximum absorption wavelength (λ_max_) of Congo red in the absence or presence of CMPs-4 and CMPs-80. The results showed that the λ_max_ of Congo red CMP complexes exhibited a significant transition into longer wavelengths in the weak alkaline solution (<0.20 M NaOH), indicating the existence of a triple helix configuration in both CMPs-4 and CMPs-80. However, when the NaOH concentration was higher than 0.2 M, the λ_max_ of Congo red CMP-80 complexes declined sharply, which might be due to the shift of space spiral structures to single flexible chains [23]. For CMPs-4, a small fluctuation was observed in the transition process of λ_max_ with the alkaline concentration increasing, which is probably because the CMP-4 fractions extracted with cold water could maintain a stable triple helical structure in the high concentration of alkaline solutions. Further research is still needed to explain the phenomenon.

### 2.6. In Vitro Antitumor Activity Analysis of Polysaccharides

**Figure 4:**
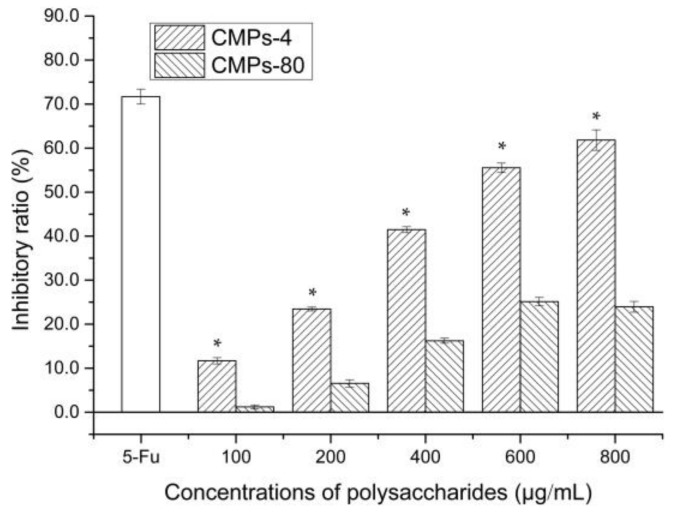
Inhibition effects of CMPs-4 and CMPs-80 by different extraction temperatures on human esophagus cancer Eca-109 cells (mean ± s, *n* = 3). Note: *, *p* < 0.05 compared with CMPs-80 under the same concentration.

An MTT assay was applied to compare the inhibitory effect of CMPs-4 and CMPs-80 towards human esophagus cancer Eca-109 cells. As shown in Figure 4, after incubation for 24 h, CMPs-4 had a significant growth-inhibitory effect on Eca-109 cells in a concentration-dependent manner, and the inhibitory ratio was found to be 11.70%, 23.44%, 41.47%, 55.54%, 61.84%, and 66.54%, respectively, with the concentrations from 100 to 1000 μg/mL. Used as the positive control, 5-Fu exhibited the strongest inhibition effect (71.70%) on Eca-109 cells with 50 μg/mL. The IC_50_ value of CMPs-4 at 24 h was calculated to be 532.9 μg/mL. In contrast, CMPs-80 showed a weaker inhibition (about <30% inhibitory rate) against human hepatoma Eca-109 cells than CMPs-4. The above data suggested that the inhibition ability of these polysaccharides was dose-dependent, and higher temperatures would reduce the antitumor activity of polysaccharides due to the damage to their structures.

**Figure 5:**
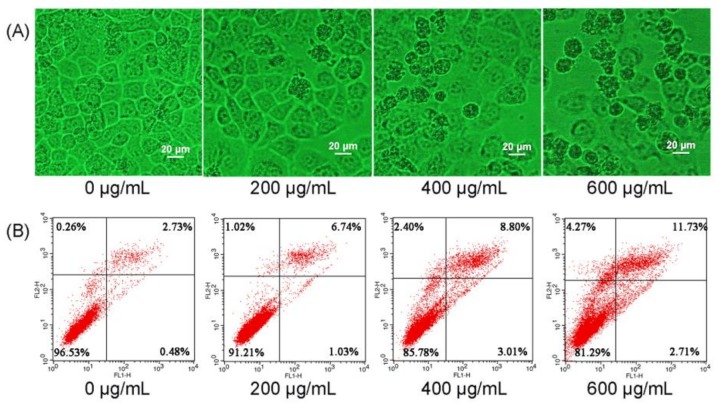

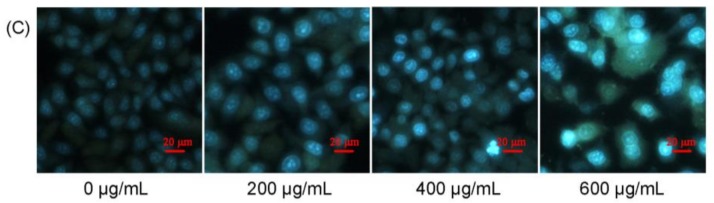
CMPs-4 induced the apoptosis of human esophagus cancer Eca-109 cells. Inverted light micrographs of Eca-109 cells showing morphological changes of cells treated with CMPs-4 for 24 h (**A**). Quantitative analysis of CMP-4-induced apoptotic cells measured using Annexin V-FITC and PI staining (**B**). Fluorescence micrographs of CMP-4-treated Eca-109 cells stained with Hoechst 33258 (**C**).

To examine whether the tumor cells’ growth suppression was caused by the induction of cell apoptosis, Eca-109 cells were treated with CMPs-4 for 24 h and the morphological changes were observed under an inverted light microscope (Figure 5A). The results showed that CMP-4-treated Eca-109 cells revealed typical apoptotic features with increasing concentrations of polysaccharides, including cell rounding and detachment, membrane blebbing, gradual loss of nuclear construction, and significant chromatin condensation, whereas these apoptosis characteristics above were not noticed in the untreated Eca-109 cells.

The CMP-4-induced cell apoptosis was further evaluated and quantified using flow cytometry analysis after Annexin V-FITC/PI staining. Seen from Figure 5B, the untreated Eca-109 cells had the highest ratio of normal cells (96.53%) without obvious cell apoptosis. When the cells were treated with CMPs-4 at the concentrations of 200, 400, and 600 μg/mL, the proportions of apoptotic cells increased from 3.21% to 7.77%, 11.81%, and 14.44%, respectively. Quantitative analysis suggested that CMPs-4 significantly increased the number of apoptotic cells in a concentration-dependent manner.

Nuclear morphology analysis can further confirm cell apoptosis by Hoechst 33,258 staining. As shown in Figure 5C, in comparison with the homogeneous and inerratic stained nuclei in untreated Eca-109 cells, CMP-4 treatment resulted in remarkable chromatin DNA condensation or nuclei fragmentation, which verified the typical characteristics of cell apoptosis.

## 3. Discussion

Esophageal cancer (EC), a highly malignant carcinoma, is estimated to be the eighth most common cancer and the sixth leading cause of cancer death worldwide [24]. Currently, chemoradiotherapy has dramatically improved the curability for unresectable malignancies compared to traditional chemotherapy or radiotherapy separately, and has been widely used to cure EC, however, serious side effects are always accompanied including tumor relapse and drug resistance [25,26]. Therefore, more and more efforts should be made to find safe and efficient bioactive substances for treating cancer. Polysaccharides, a kind of biological response modifier, have been extensively used as preventive and therapeutic agents for cancer because of their relatively low toxicity and effective antitumor activities. As reported, the polysaccharides’ bioactivities were closely correlated to their structural features, including monosaccharide composition, molecular weight, functional groups, chain conformation in solution, and so on [27,28]. 

Temperature, as a critical parameter, influences various biological activities in animals and plants [29]. Currently, hot water (80–100 °C) is the most common polysaccharide extraction solvent, but the bioactivities may be reduced under high extractive temperatures due to degradation [30] and oxidation [31]. It has been reported that cold-water-extracted (4 °C) polysaccharides from *Astragalus membranaceus* exhibit the stronger antitumor and immunoregulation activities, with minimal side effects due to the degradation of active constituents by higher temperature [28]. In the present study, the differences of structural characteristics between polysaccharides from *C. militaris* with two extraction temperatures (4 and 80 °C) were deeply researched. Results showed that monosaccharides’ molar ratio and the molecular weight distribution of these two polysaccharides exhibited significant discrepancies, where CMPs-80 had higher proportions of rhamnose and galactose, but a lower proportion of glucose. In addition, the CMPs-80 showed a higher molecular weight (about 308 kDa) with a relative high percentage area (62.46%), while CMPs-4 had a component about 188 kDa with a percentage area (53.23%). These results indicated that higher extraction temperature could degrade the polysaccharide mainly composed of glucose and increase the dissolution rate of polysaccharides consisting of rhamnose and galactose. The IR spectrums, specific optical rotation analysis, and conformational characteristics showed no significant difference between these two polysaccharides.

Apoptosis is an important method for multicellular lives to maintain homeostasis and a key survival process for hosts when it occurs only tumor cells [32,33]. Characteristic morphological changes can be found in apoptotic cells, such as membrane bubbling, karyopyknosis, and chromatin condensation [34,35]. In this study, CMPs-4 exhibited much stronger inhibitory effects on Eca-109 cells and induced the cells to apoptosis identified via the above characteristics, which indicated that higher temperatures could destroy the structure of polysaccharides and reduce their antitumor activity. Considering the relationship of the structure characteristics and antitumor effect, we can conclude that the component of 188 kDa in the polysaccharide from *C. militaris* is responsible for the antitumor activity, which need to be further researched in the future.

## 4. Materials and Methods

### 4.1. Plant Material and Chemical Reagents

*C. militaris* were obtained from the incubation base of industry-university-research cooperation in YiLi Normal University (Sinkiang, China), and were first washed with deionized water thoroughly to remove all dirt and other contaminants. The *C. militaris* were dried to constant weight in an oven at 50 °C, screened through an 80 mesh sieve to obtain the homogeneous powder sample, and finally stored under suitable conditions for subsequent study. Fetal calf serum (FCS) was from Hangzhou Sijiqing Co. (Zhejiang, China). Annexin V-FITC/PI Apoptosis Detection Kits were purchased from Beyotime Biotechnology Co. (Shanghai, China). 5-fluoro-2,4(1h, 3h)pyrimidinedione (5-Fu), 3-(4,5-dimethyl-2-thiazolyl)-2,5-diphenyl-2-*H*-tetrazolium bromide (MTT), and dimethyl sulfoxide (DMSO) were purchased from Sigma-Aldrich (St. Louis, MO, USA). Other chemicals and agents were of analytical grade.

### 4.2. Extraction of Crude Polysaccharides

The extraction procedure was carried out using water extraction and alcohol precipitation. The dried *C. militaris* powder (50 g) was extracted with distilled water (1:30 ratio of solid to liquid, *w/v*) at either 4 or 80 °C for 4 h. After extraction, the water extracts were filtered and centrifuged (4500 rpm, 15 min) to remove the insoluble fractions. The collected supernatant was concentrated by freeze-thaw method and then precipitated by the addition of 4 volumes of absolute ethanol overnight at 4 °C. The precipitated polysaccharides were obtained after centrifugation at 4500 rpm for 10 min, and washed using anhydrous ethanol and acetone alternately 3 times to remove the lipids and pigments thoroughly. Afterwards, the crude samples were re-dissolved in deionized water, and the protein was removed according to the Sevag method with 1-butanol and chloroform (1:4, *v*/*v*) for 5 times [36]. Finally, the mixture was concentrated to remove the Sevag reagents, dialyzed (*M*_w_ 1000) in distilled water for three days to exclude the salt and low molecular weight sugars, and lyophilized to get the *C. militaris* polysaccharides (CMPs) for further study.

### 4.3. Determination of Carbohydrate and Protein Content

The contents of carbohydrate in the CMPs-4 and CMPs-80 were determined according to the phenol-sulfuric acid method using d-glucose as a standard [37]. The protein contents of the two samples were estimated by the Bradford’s method using bovine serum albumin (BSA) as the standard [38].

### 4.4. Monosaccharide Composition Analysis of CMPs

The monosaccharide composition of the polysaccharide samples was determined after acid hydrolysis and derivatization using GC (Shimadzu, Kyoto, Japan) according to the procedures described previously [27]. In brief, the dried samples (5.0 mg) were hydrolyzed with 2 mL of TFA (2 M) in the sealed tubes at 120 °C for 4 h. After TFA was removed, the hydrolysates containing myo-inositol as their internal standard were further acetylated and thoroughly dissolved in dichloromethane for GC analysis. In addition, l-rhamnose, d-Arabinose, d-Xylose, d-Mannose, d-Glucose, and d-Galactose were also derivatized as the standards to identify the monosaccharide components of the samples by comparing the retention times.

### 4.5. Molecular Weight Distribution of CMPs

The molecular weight distribution of CMPs-4 and CMPs-80 was performed by HPGPC (Agilent-1200, Agilent, Santa Clara, CA, USA) equipped with a TSK-gel G4000 PWxl column (7.8 mm × 300 mm, column temperature 30 °C) and Refractive Index Detector (RID, detecting temperature 35 °C) based on the approaches described previously [39]. Twenty μL of sample solution was injected and run with distilled water as mobile phase at a flow rate of 0.6 mL/min. The molecular weight distribution of polysaccharides was calculated according to the calibration curve established using T-series Dextran standards (T-10, T-40, T-70, T-500 and T-2000).

### 4.6. FT-IR Spectrum Analysis of CMPs

The characteristic groups of the polysaccharide samples were identified using FT-IR. A total of 0.7 mg of dried polysaccharide samples were blended with 150 mg of dry KBr powder, followed by pressing into a KBr disk for further research. The IR spectra was collected in the range of 4000–400 cm^−1^ on a FT-IR spectrophotometer (Bruker VECTOR-22, Karlsruhe, Germany).

### 4.7. Determination of Specific Optical Rotation

The digital automatic polarimeter (Precision & Scientific Instrument Co., Ltd., Shanghai, China) was applied to determine the optical rotation of the polysaccharide samples. The 10 mg CMPs-4 and CMPs-80 were dissolved with 10 mL distilled water, respectively, and transferred into the polariscope tubes. The optical rotation of the samples was read directly with an automatic polarimeter at the designated temperature (20 ± 0.1 °C). The specific optical rotation was calculated according to the following equation:

$${\left[\alpha \right]}_{\lambda }^{t}=\frac{\left[\alpha \right]}{L\times C}$$

where α is the optical rotation, *t* is the detecting temperature, λ is the wavelength of light source (λ = 589 nm), *L* is the length of polariscope tube, and *C* is the polysaccharide solution concentration (g/mL).

### 4.8. Conformational Analysis of CMPs

The conformation of the CMPs-4 and CMPs-80 was evaluated following the method reported by Su et al. [22] with little modification. Two milliliters of 0.5 mg/mL sample solution were mixed with 2 mL of 50 μM Congo red solution and a different volume of 1 M NaOH solution to make the final concentrations of NaOH solutions of 0, 0.05, 0.10, 0.15, 0.20, 0.25, 0.30, 0.35, and 0.40 M. The reaction of the mixtures was performed for 10 min at room temperature, and subsequently the absorbance was scanned from 400 to 600 nm using an ultraviolet spectrophotometer (Infinite M200 PRO, Tecan, Crailsheim, Germany). A same volume of deionized water as the previously mentioned polysaccharides solution was used as a blank control.

### 4.9. In Vitro Antitumor Activity

#### 4.9.1. MTT Assay

The viability of Eca-109 cells was evaluated in vitro using an MTT assay as previously described [40], with minor modifications. Eca-109 cells were cultivated to logarithmic phase with an RPMI 1640 medium containing 10% FBS at 37 °C in 5% CO_2_, and 100 μL of cell suspension (1× 10^5^ cells/mL) was gently seeded into 96-well cell culture plates and incubated overnight. Subsequently, Eca-109 cells were subjected to different concentrations of sterile polysaccharide solutions (0, 100, 200, 400, 600, and 800) and incubated at 37 °C in a humidified 5% CO_2_ incubator for 24 h, followed by the addition of 20 µL of MTT (5 mg/mL in PBS) into each well. After 4 h of incubation, the supernatant was removed by centrifugation at 1000 rpm for 10 min and the precipitated formazan was dissolved in 150 µL/well of DMSO. 5-Fu was used as the positive control. The absorbance value was detected at 570 nm using a microplate reader (Model 680, Bio-Rad, Hercules, CA, USA). The inhibition effect of the polysaccharides on Eca-109 cells was calculated as follows: Inhibitory ratio (%) = (1 − *A*_Treated_/*A*_Control_) × 100.

#### 4.9.2. Morphologic Observation

Eca-109 cells (8 × 10^4^ cells/mL) were inoculated into 6-well plates and treated with different concentrations (0, 200, 400, and 600 μg/mL) of CMPs-4. After 24 h, the morphological changes of Eca-109 cells were observed under an inverted optical microscope (ECLIPSE Ti, Nikon, Tokyo, Japan).

#### 4.9.3. Annexin V/PI Double-Staining 

Eca-109 cells (8 × 10^4^ cells/mL) cultured in 6-well plates were treated with an Annexin V/PI Apoptosis Detection Kit according to the directions. After treatment with the designated concentrations (0, 200, 400 and 600 μg/mL) of CMPs-4 for 24 h, the cells were collected and stained with the Annexin V-FITC/PI apoptosis detection kit following the manufacturer’s manual. In brief, the harvested cells were incubated with 100 μL of a 1 × binding buffer containing 5 μL Annexin V-FITC and 5 μL propidium iodide (PI) for 10 min at room temperature in the dark. The stained cells were detected immediately by flow cytometry (BD FACSCallibur, BD, Franklin Lakes, NJ, USA).

#### 4.9.4. Hoechst 33258 Staining

Eca-109 cells (8 × 10^4^ cells/mL) were seeded into the 6-well cell culture plates and exposed to various concentrations of CMPs-4 (0, 200, 400 and 600 μg/mL) for 24 h. Subsequently, the cells were collected, washed with cold PBS, and stained with Hoechst 33258 solution at room temperature for 15 min. After that, the stained cells washed in PBS and observed by inverted fluorescence microscope (ECLIPSE Ti, Nikon, Tokyo, Japan).

### 4.10. Statistical Analysis

All values were expressed as the mean ± standard deviation (S.D.) and the statistical significance of differences was determined using the student’s t-test and one-way analysis of variance (ANOVA). The data was considered significant at *p* < 0.05. All statistical analysis was carried out using SPSS 19.0 (SPSS Inc., Chicago, IL, USA).

## 5. Conclusions

In conclusion, we extracted the polysaccharides from *C. militaris* with water under two different temperatures (4 °C, CMPs-4; 80 °C, CMPs-80), and researched the relationship between the structural characteristics and antitumor effects. Our results showed that higher extraction temperatures could degrade the polysaccharide with 188 kDa, mainly composed of glucose, and increase the dissolution of polysaccharide with 308 kDa, mainly consisting of rhamnose and galactose. The in vitro antitumor test showed that CMPs-4 possessed stronger antitumor activity than CMPs-80, which could significantly inhibit the proliferation of Eca-109 cells via inducing their apoptosis. This study provides a novel extract method to obtain polysaccharides with higher bioactivity and a theoretical basis for the application of CMPs-4 in food and medical industries.

## Figures and Tables

**Table 1:** Contents of total sugar and protein, monosaccharide composition and their molar ratio of CMPs-4 and CMPs-80 (mean ± s, *n* = 3).

Samples	Total sugar(%)	Protein content(%)	Monosaccharide component					
			rhamnose	arabinose	xylose	mannose	glucose	galactose
CMPs-4	87.63 ± 3.58	0.82 ± 0.05	0.24	0.57	0.48	1.00	12.41	1.63
CMPs-80	85.42 ± 3.49	1.21 ± 0.06	3.98	0.62	0.42	1.00	6.70	3.18

**Table 2:** Molecular weight distribution of CMPs-4 and CMPs-80 (mean ± s, *n* = 3).

Samples	*R*_t_ (min)	*M*_w_ (kDa)	Relative content (%)
CMPs-4	9.241	188.20 ± 14.32	53.23 ± 1.54
	14.096	2.50 ± 0.57	46.76 ± 2.88
CMPs-80	8.688	307.91 ± 18.98	62.46 ± 0.66
	14.211	2.26 ± 0.22	34.81 ± 2.52

*M*_w_: Molecular weight; *R*_t_: Retention time.
